# Applying Fishers' Ecological Knowledge to Construct Past and Future Lobster Stocks in the Juan Fernández Archipelago, Chile

**DOI:** 10.1371/journal.pone.0013670

**Published:** 2010-11-05

**Authors:** Tyler D. Eddy, Jonathan P. A. Gardner, Alejandro Pérez-Matus

**Affiliations:** 1 School of Biological Sciences, Centre for Marine Environmental and Economic Research, Victoria University of Wellington, Wellington, New Zealand; 2 Victoria University Coastal Ecology Laboratory, School of Biological Sciences, Victoria University of Wellington, Wellington, New Zealand; University of Glamorgan, United Kingdom

## Abstract

Over-exploited fisheries are a common feature of the modern world and a range of solutions including area closures (marine reserves; MRs), effort reduction, gear changes, ecosystem-based management, incentives and co-management have been suggested as techniques to rebuild over-fished populations. Historic accounts of lobster (*Jasus frontalis*) on the Chilean Juan Fernández Archipelago indicate a high abundance at all depths (intertidal to approximately 165 m), but presently lobsters are found almost exclusively in deeper regions of their natural distribution. Fishers' ecological knowledge (FEK) tells a story of serial depletion in lobster abundance at fishing grounds located closest to the fishing port with an associated decline in catch per unit effort (CPUE) throughout recent history. We have re-constructed baselines of lobster biomass throughout human history on the archipelago using historic data, the fishery catch record and FEK to permit examination of the potential effects of MRs, effort reduction and co-management (stewardship of catch) to restore stocks. We employed a bioeconomic model using FEK, fishery catch and effort data, underwater survey information, predicted population growth and response to MR protection (no-take) to explore different management strategies and their trade-offs to restore stocks and improve catches. Our findings indicate that increased stewardship of catch coupled with 30% area closure (MR) provides the best option to reconstruct historic baselines. Based on model predictions, continued exploitation under the current management scheme is highly influenced by annual fluctuations and unsustainable. We propose a community-based co-management program to implement a MR in order to rebuild the lobster population while also providing conservation protection for marine species endemic to the Archipelago.

## Introduction

As a response to reports of declining and unsustainable fisheries worldwide [1]–[5] there has been much debate among conservationists, fisheries biologists and fisheries managers [6] about the best means to balance sustainable exploitation with conservation of biodiversity and ecosystems. Proposed solutions include, but are not limited to, ecosystem-based management, MRs and other forms of fishery closures, incentives, co-management, total allowable catch (TAC) and individual transferable quotas, reductions in fishing fleet capacity and changes in gear regulations [7]–[9]. Elsewhere it has been suggested that the tools for effective management of fish stocks are already available and that fishery science is sound, but that recommended harvest limits are rarely implemented as policy because of lobbying by stakeholders [10]. We examine co-management strategies in Chile where the stakeholder group that most often objects to fisheries regulations, fishers, has taken a major role in the management of their livelihood. In the absence of information about the response of lobster to MRs in Chile, we examine the potential of MRs for fisheries management in the Juan Fernández Archipelago using observations from New Zealand. We then investigate the effects co-management, MRs and traditional fisheries management tools for their effectiveness to rebuild an overexploited Chilean lobster (*Jasus frontalis*) fishery as well as promote conservation values and ecosystem protection.

### Marine Reserves and Co-Management

In Chile and New Zealand, MRs are implemented for conservation purposes, but may produce indirect benefits for fisheries because they have been shown to increase the size, abundance and biomass of many fished species, including the New Zealand lobster, *Jasus edwardsii* [11]–[14], to safe-guard against fishery-associated handling disease [15], and to increase population-specific egg production rates because larger lobsters produce disproportionately more eggs than smaller lobsters [11]. However, while the area of the MR may benefit from a reduction in fishing pressure, adjacent areas may not. For example, the implementation of a MR often displaces fishing effort, resulting in greater fishing effort per unit area outside the MR [16]–[19]. A concern often voiced by fishers is that if the MR does not benefit the region by providing lobsters via spillover, then CPUE will be lower in areas adjacent to MRs. However, this need not be the case as CPUE at locations adjacent to a MR and locations further afield may be similar, although the catch made surrounding the MR may be represented by fewer, larger lobsters resulting in a similar amount of profit per trap haul (e.g., [20]). Lobsters protected by a MR as small as 400 ha have increased in density, with larger adults making periodic movements across the MR boundary where they “spillover” to the fishery [20], a phenomenon influenced by the position of MR boundaries in relation to rocky reef habitat because lobsters are less likely to cross soft sediment habitat [21].

Co-management between fishery managers and fishers has resulted in several benefits in Chile, including (1) it changes the nature of fishing as fishers become stewards of the resource and catches become more predictable [22], [23]; (2) compliance is greater in a community-managed system where local stakeholders have a vested economic interest in the welfare of the resource [24]; (3) it increases the conservation ethic of fishers with greater conservation-oriented attitudes correlated to a longer involvement with co-management [24], [25]; (4) it increases biodiversity in co-managed areas [25] and (5) it may act as a bridge to implement further conservation actions such as MRs. However, in order for a system of co-management to experience high compliance, fishers need be an integral part of the management process which strives to achieve goals set by community [26]–[31]. A bioeconomic evaluation of co-management needs to include social, economic and biological components; without all of these elements the system becomes oversimplified [32].

In order to evaluate sustainable fishery management options, it is necessary to determine the current level of stock exploitation. Comparison of stock biomass at present time to “virgin” biomass (biomass under an exploitation rate of 0) indicates how exploited a stock has become [33]. Stocks that are fished below the biomass that produces maximum sustainable yield (BMSY) are less productive and it maybe desirable from both economic and ecological perspectives to rebuild the stock to a historic, more abundant state. However, it may be difficult to determine if a stock has been fished below BMSY if there is a lack of information about historic stock abundance. This can lead to what has been dubbed “the shifting baseline syndrome” resulting in a distorted view of what is “virgin” biomass [34]. In the absence of stock abundance estimates over time, alternate techniques employing historical knowledge from non-scientific sources are needed to place the current state of stock abundance in a larger context [7], [35]–[37]. Historic accounts and fishers' ecological knowledge (FEK) are information sources that can provide insight into changes in stock abundance on inter-generational time scales and prior ecosystem states [7], [38].

### The Chilean Juan Fernández Lobster Fishery

**Figure 1:**
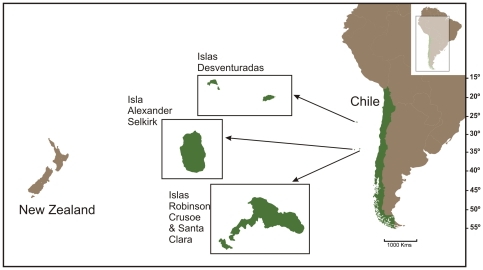
Location of Juan Fernández Archipelago and Islas Desventuradas in relation to Chile and New Zealand. Islas Robinson Crusoe, Santa Clara and Alexander Selkirk collectively make up the Juan Fernández Archipelago.

**Figure 2:**
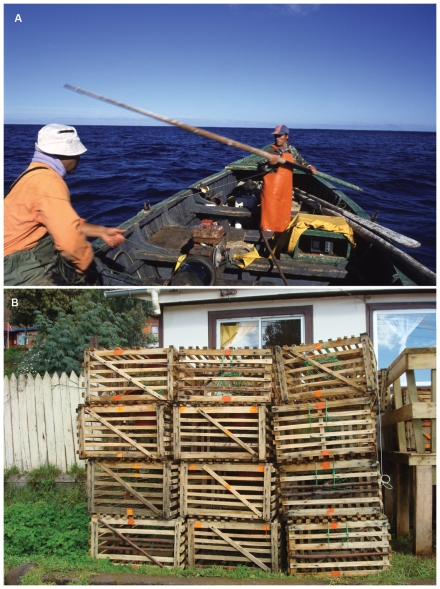
Lobster fishing gear on the Juan Fernández Archipelago. **A** - Technology consists of a wooden boat, gas-powered winch, depth finder and outboard engine. Photograph by Alejandro Perez-Matus. **B** - Wooden lobster traps. Photograph by Fabian Ramírez.

Our study site, the Chilean Juan Fernández Archipelago, is located in the south Pacific Ocean (33° 37′S, 78° 51′ W), 700 km west of the port city of Valparaíso (Figure 1). The volcanic islands that make up the archipelago (Robinson Crusoe, Santa Clara and Alexander Selkirk) display a high degree of endemism in both terrestrial and marine environments [39]–[41]; this applies to the lobster, *Jasus frontalis*, found only on the Juan Fernández Archipelago and the Islas Desventuradas (Figure 1). The lobster fishery is the main source of economic revenue for fishers inhabiting the Archipelago. The decline in lobster abundance and the associated change in its natural distribution are documented in historic accounts [42]–[45]. Bahía Cumberland is the main fishing port of the Juan Fernández Archipelago from which approximately 180 fishers operate approximately 40 boats to fish at Islas Robinson Crusoe and Santa Clara [46]. Additionally, seasonal fishing camps are set-up on Alexander Selkirk Island. Wooden boats between 8 to 10 m in length are powered by an outboard motor, and sometimes fitted with a winch and depth finder (Figure 2A; [47]). Lobster fishing practices using wooden traps (1.35 m by 0.78 m by 0.37 m; [47]) have remained relatively traditional (Figure 2B).

The current lobster management regulations include a seasonal closure from May 15^th^ until September 30^th^, a minimum cephalothorax harvest size of 11.5 cm and no capture of egg-carrying females, however there are no catch limits. An informal management system exists whereby location of trap placement is governed by a complex, highly structured system with high compliance [48]. Because it is based on the use of traps, the fishery itself is selective, but several finfish species are caught for bait via other methods, resulting in the harvest of approximately 150 kg of fish per day (personal communication with local fishers). The current lobster fishing effort on Islas Robinson Crusoe and Santa Clara is concentrated in the farthest reaches of the archipelago in relation to the population centre and main port, Bahía Cumberland [48]. Fishers who camp on Isla Alexander Selkirk (the most isolated region of the Archipelago) report a much higher CPUE [49]. At present, STIPA Juan Fernández (the Juan Fernández fishers' syndicate) is a fishers' union concerned with the marketing and management of lobster with a mandate for the conservation and sustainable management of marine biodiversity within the archipelago. The lobster fishery on Juan Fernández is classified by the Chilean government as “artisanal” which gives exclusive fishing rights to registered fishers on the archipelago from land to five nautical miles offshore and prevents new fishers from entering the fishery.

### Lobster Stocks: Past and Future

Our research focuses on Islas Robinson Crusoe and Santa Clara due to limited access to, and lack of availability of information for, Isla Alexander Selkirk. Combining FEK, underwater observations and collection of historic, government and scientific information for Islas Robinson Crusoe and Santa Clara, our aims are three-fold. First, we estimate baselines of lobster biomass over 400+ years of human fishing activity on the archipelago which has led to the current lowest recorded catches in history. Second, we develop a bioeconomic fishery model to describe the dynamics of lobster abundance and the catch record throughout the last century using biological parameters from the closely related lobster species, *Jasus edwardsii*, in the absence of such biological data for *J. frontalis*. Third, we use the bioeconomic model to predict how differing management strategies will impact both lobster abundance and fishery catch over the next 40 years to restore stocks and promote a sustainable fishery. Overall, we describe a 500-year period of lobster exploitation and assess the trade-offs between catch and conservation in the social context of the artisanal fishing community at the Juan Fernández Archipelago. We demonstrate that this technique of reconstructing baselines utilising biological, historic and social information is a powerful tool to understand the relationship between prior and current stock states when considering future management options for conservation and sustainable exploitation of coastal resources.

## Methods

### Reconstructing Baselines

We refer to the period of early human exploitation of the marine resources of the Juan Fernández Archipelago before large numbers of lobsters were removed from the population as the “virgin” period (1574–1898). As the intertidal zone and shallow subtidal depths were fished of lobsters [42]–[45], effort moved to deeper waters, which we term the “historic” period (1898–1930). The fishery catch record begins in 1930 for all landings of *Jasus frontalis* in Chile (including Isla Alexander Selkirk and the Islas Desventuradas). The proportion of catch from Islas Robinson Crusoe and Santa Clara was estimated to be 65% (±5% standard deviation) from 1972–1983 [50] and 49% for the 1996–1997 season [49]. We used these values to model a catch record for Islas Robinson Crusoe and Santa Clara which show landings varying by as much as 46 tonnes between successive years (e.g., 1942 versus 1943). Although highly variable, the average catch remained stable at approximately 60 tonnes per year until 1967. We call 1930–1967 the “fishing” period. From 1967 until 1982, catches declined despite evidence of increasing fishing effort [50], after which they leveled off and reached a new average catch of approximately 20 tonnes per year. Lobster catch for all of the island groups was declared to be 1 tonne in 2004 when the fishery was closed by the Chilean National Fishery Service (SERNAPESCA) for one season to allow stocks to recover, resulting in the 2005 and 2006 seasons producing the highest catches in 30 years. The last year for which we have catch data is 2008. Based on the low catch and increasing effort we define the years 1967–2008 as the “over-fishing” period. We identify these four designations in the history of lobster fishing on the Juan Fernández Archipelago because each period represents a different state for lobsters in terms of biomass. We use information from these separate periods to facilitate the calculation of average baselines and thereby model development for the “fishing” and “over-fishing” periods, to allow us to investigate how alternative management strategies might influence what we call the “future of fishing” period from 2008–2050.

We used the most recently published stock assessment and composition of catch by depth data [49], [51] to estimate the lobster biomass during the “over-fishing” period. In the 1996–97 fishing season, the Robinson Crusoe – Santa Clara stock was estimated to be approximately 70 tonnes [51] and the reported catch averaged over those two years was 34 tonnes [46] indicating that approximately 50% of available stock was harvested [49]. We used this value of 50% to calculate lobster biomass for the average catch made during the “over-fishing” period in which most traps were set in water between 112 and 165 m deep [49]. For the “fishing” period, we assumed that catch represented a smaller proportion of total stock biomass because CPUE was higher during this period [50]. Based on the historic accounts of biomass and depth of “best catch” [43]–[46], CPUE [49], [52], and area of habitat by depth, we estimated lobster biomass for the “historic” and “virgin” periods.

### Quantifying Spatial and Temporal Trends

Conversations with elders of the Juan Fernández Archipelago fishing community (FEK; [38]) provided information about spatial changes in lobster abundance and fishing effort throughout recent history that we could not find in government or literature sources. These fishers have been fishing on the Juan Fernández Archipelago for as many as 40 years, providing information dating back to 1967 in some cases, corresponding to the transition from the “fishing” to the “over-fishing” period. Anecdotal information about the spatial and temporal distributions of fishing effort, catches and lobster abundances throughout the archipelago were recorded as either “high”, “moderate” or “low”. This information was used to understand how lobster abundance, catch and effort have changed in the archipelago during the last 40 years and was used during development of spatial dynamics for the model described below.

**Figure 3:**
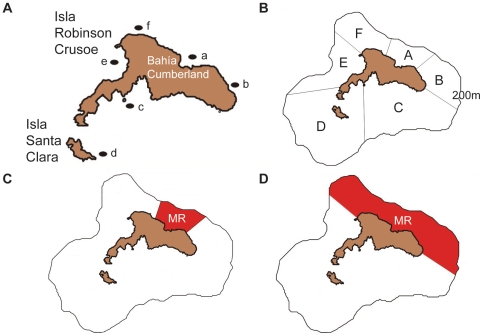
Map showing underwater survey locations, spatial regions and MR locations. **A** – Map of Islas Robinson Crusoe and Santa Clara showing the main fishing port of Bahía Cumberland and underwater survey locations (a–f; see Table 1 for names). **B** – Spatial regions used in model scenarios (A–F) with 200m depth contour. **C** – Location of 10% MR used in model scenarios. **D** – Location of 30% MR used in model scenarios.

In order to quantify fishers' observations of current lobster abundance distributions at shallow depths (<30m), we conducted underwater surveys of invertebrate abundance during the months of September and October, 2007 (*manuscript in review*). We selected sites around Isla Robinson Crusoe and Isla Santa Clara to sample separate regions of the archipelago with different subtidal habitats and wave exposure (Figure 3A). Wave-exposed sites located on the western side of the archipelago are characterised by vertical walls, big boulders and caves, whereas eastern sites are more gradual in slope, highly eroded and characterised by sand, small boulders and cobble. At each site, a 0.25 m^2^ quadrat (50×50 cm) was placed at 4 m intervals on both sides of a 20 m transect (10 quadrats per transect) recording the abundance of invertebrate species including lobster. Between six and ten transects were completed at each site (mean = 6.5±2.3 standard deviation; 39 total) based on depth and weather conditions. Transects were conducted perpendicular to the shore to survey a range of depths at each site. Lobster abundance per site is expressed as a percentage of total abundance at all sites, standardized for area surveyed.

### Bioeconomic Fishery Model

We have employed a Schaefer biomass dynamic fishery model ([53]; equation 1), an economic and behavioural fishery model ([54]; equation 2) and a biological movement model in a spatial context ([54]; equation 3).
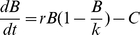
(1)

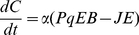
(2)

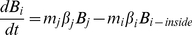
(3)These models are combined to produce a bioeconomic fishery model (equation 4), described by the terms: *B_i_* = biomass of lobsters in region *i*, *t* = time, *r* = intrinsic rate of population growth, *k*_i_ = carrying capacity in region *i*, *C_i_* = catch in region *i*, *α* = rate at which changes in fishing effort take place, *P* = price, *q*_i_ = effectiveness of fishing effort (catchability) in region *i*, *E_i_* = fishing effort in region *i*, *J_i_* = travel cost to fishing grounds for region *i*, *m_i_* = movement rate of lobster biomass in region *i*, 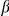 *_i_* = uniform distribution variable in region *i*, and *ε* = annual variability.
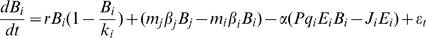
(4)We use the term *j* to refer to regions neighboring region *i* in a spatial context. The uniform distribution probability (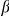) is a randomly generated value between 0 and 1 to simulate the probability of lobster biomass moving to a neighbouring region, given a specific movement rate and lobster density for the region of origin. We chose the Schaefer biomass dynamic model (equation 1) because of the absence of age-structured data for the time scale of the fishery catch record we examined [55].

We have incorporated the economic terms price (*P*) and cost (*J*) in the model (equations 2 and 4) to reproduce observations by fishers (FEK) of changes in stock abundance both spatially and temporally. Using these terms in the model allows for areas closest to Bahía Cumberland (the main port) to be fished first given sufficient lobster density. We use the *α* term (responsiveness to changes in stocks) in order to model fishers' behaviour to changes in stocks. Following a season with a lower catch, fishers fish less in order to maintain stock biomass, a strategy employed in Chilean MEABRs [22], [24], [56]: we refer to this behaviour as “stewardship”. The model assumes that fishing technology remains constant during each period (fishing, over-fishing, future of fishing) which is defined as catchability (*q*) in the model.

By designating separate spatial areas (Figure 3B; *i* and *j*), we are able to input region-specific lobster biomass (*B_i_*), fishery catch (*C_i_*), travel cost (*J_i_*) and movement rates (*m_i_*), as well as simulate areas designated as MRs (no-take) to predict associated density-dependent spillover effects. Six spatial areas were designated based on lobster abundances and habitat classifications observed during underwater surveys (Figure 3A and B), locations of historical and current lobster abundance and fishing efforts (FEK), and accessibility to different regions of the archipelago. We have allowed for the movement of lobsters between neighbouring regions in the model according to their density and movement rate (*m_i_*, *m_j_*), multiplied by a uniform distribution probability (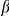 *_i_*, equation 4). This feature of the model allows for “spillover” of lobsters from areas of higher density to areas of lower density, a phenomenon that has been documented in New Zealand for *Jasus edwardsii* [20], [21], a species which shares many biological characteristics with *Jasus frontalis* [57]–[62]. As a consequence, fishing effort changes spatially in response to lobster biomass (*Bi*), cost of travel (*J_i_*) and fishers' responsiveness to changes in lobster biomass (*α*; equation 4).

### Parameter Estimation

Given the large timescale we are working with and the limited amount of data, we used a variety of sources and techniques to estimate parameters ([57]–[62]; Table 1). Intrinsic rate of increase (*r*) was estimated from two sources; first, from a literature value for *Jasus frontalis* [50], and second, using data for the recovery of 14 *Jasus edwardsii* populations following the reduction of fishing pressure in New Zealand after MR implementation [14]. Both values were calculated for a highly exploited stock biomass indicating that they should be accurate values of growth for our model and only varied by 0.06% [55]. Biomass dynamic models are sensitive to intrinsic rate of population growth (*r*) at low biomass [55], however we have a high confidence in our value for the model due to the similar values given by empirical evidence for both *Jasus edwardsii* and *Jasus frontalis* [14], [50]. Carrying capacity (*k*) was estimated using historic accounts of lobster density [42], [43] and extrapolated to area of suitable habitat in each *i* region. Initial lobster biomass was estimated using the catch record, reports of CPUE [49], [50], stock assessments [51], FEK, and historic accounts. Effort was determined spatially using accounts of FEK and data from the 1972–1983 period [50] and the 1996–1997 season [49]. Movement rate was calculated from tagging and MR spillover studies for *Jasus edwardsii* [20], [63], [64] and adjusted for the area of each of the six spatial areas (smaller area = greater chance of emigration). Price of lobster and travel cost per unit lobster were chosen such that patterns of fishing predicted by the model were similar to those reported by the fishers over time. For each of the “fishing” and “over-fishing” periods, four free parameters (*α*, *ε*, *P* and *q*) were estimated by minimising residual sums of squares (RSS) in comparison to observed data [65] and were used for the “future of fishing” period.

**Table 1:** Model parameters and initial conditions for the three periods; Fishing, Over-fishing and Future of fishing.

State variables, Parameters and units	Fishing Period (1930–1967)	Over-fishing Period (1967–2008)	Future of Fishing Period (2008–2050)
*Lobster population*			
growth rate – *r*	0.0672	0.0672	0.0672
carrying capacity – *k* (kg)	400 000	400 000	400 000
initial biomass* – *B* (kg)	200 000	116 000	61750
movement rate* – *m_i_*	0.04–0.125	0.04–0.125	0.04–0.125
uniform distribution variable - 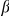	0–1	0–1	0–1
*Fishery catch*			
initial effort* – *E* (number of fishing trips)	805	2414	1207–2414
stewardship - *α*	0.001	0.0012	0.001–0.01
catchability – *q*	0.00001	0.00003	0.00003
price – *P*	20	20	20
cost – J_i_*	4–10	5–10	5–10
annual variation - *ε* (standard deviation)	coswave (30 000, 4)	sinwave (20 000, 4)	sinwave (20 000, 4)

State variables and parameters that were spatially resolved are indicated by *.

### Model Validation and Prediction

We confronted competing bioeconomic models composed of varying numbers of the four free parameters (*α*, *ε*, *P* and *q*) with the observed catch data for the *a priori* defined “fishing” and “over-fishing” periods. Model iterations were integrated using the Runge-Kutta 4 method with a time step of 0.125 years and were run for 37 years for the “fishing” period (1930–1967), 41 years for the “over-fishing” period (1967–2008) and 42 years for the “future of fishing” period (2008–2050) using STELLA software [54]. We used an Akaike Information Criterion (AIC) approach [66] to assess competing model performance:
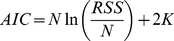
(5)where *N* is the sample size, *K* is the number of model parameters and *RSS* is the residual sums of squares. Lower *AIC* values indicate a better level of model support [66].

The “future of fishing” (2008–2050) model employed scenarios with regions designated as MRs (closed to the fishery), fishing effort reduction (ER) and stewardship, as well as “business as usual”, indicating no change in management practice. The “future of fishing” model utilised the same optimised parameters (*α*, *ε*, *P* and *q*) as the “over-fishing” period. Eight different scenarios were run for 42 years with variable amounts of fishing effort and area, with or without stewardship of catch. The 10% MR (10% of fishing grounds closed) is centered in Bahía Cumberland (Figure 3C), which was suggested by lobster fishers to be the best location because it is the area most depleted in abundance and most easily enforced and monitored by “the eyes of the village”. The placement of the 30% MR (30% of fishing grounds) is centered in Bahía Cumberland as previous, but extended to the east and west to include El Francés and Sal si puedes (Figure 3D). These three regions are the least fished, with the lowest number of traps set throughout the archipelago, that is, 15.6% of all traps in 30% of the area [49].

## Results

### Historic Baselines

Visitors to the Juan Fernández Archipelago in the 1700's found that lobsters were “… in such abundance near the water's edge (of Isla Robinson Crusoe) that the boat-hooks often struck into them, in putting the boats to and from the shore” [42] and were “ … found in such quantities that the fishermen have no other trouble than to strew a little meat upon the shore, and when they come to devour this bait, as they do in immense numbers, to turn them on their backs with a stick…” [43]. Almost one hundred years later, lobsters “… were fished at depths of 7 to 14 m …” ([44], p. 6), while fifty years after this “… the best catch is made in depths from 40 to 80 m …” ([45], p. 178). The most recent study during the 1996–1997 season found that the highest number of lobsters per trap occurred between depths of 112 to 165 m at Islas Robinson Crusoe and Santa Clara with a CPUE of 10 lobsters per trip compared to 32 per trip at Isla Alexander Selkirk, and 174 per trip at Islas Desventuradas [49]. Historic lobster abundance estimates in the intertidal and shallow subtidal zones described by Walter [42] and Molina [43] are substantially different from those described by Albert [44] and Skottsberg [45]. FEK and underwater observations show that the majority of lobster abundance is currently concentrated in the farthest reaches of the Archipelago (Table 2).

**Table 2:** Fishers' ecological knowledge recorded by region from lobster fishers and results of underwater survey for *Jasus frontalis* (% of total abundance) at sites on the Juan Fernández Archipelago.

Site or region	Previous abundance	Current abundance	Previous fishing effort	Current fishing effort	% of total abundance
A - Bahía Cumberland	Moderate	Low	High	Low	3.1
B - El Francés	Moderate	Low	Moderate	Low	16.1
C - Los Chamelos	High	Moderate	Low	Moderate	6.3
D - Santa Clara	High	Moderate	Low	High	61.9
E - El Cernícalo	High	Moderate	Low	Moderate	9.4
F - Sal si puedes	Moderate	Low	Moderate	Low	3.1

Refer to Figure 3 (panels A & B) for location of sites and regions.

**Figure 4:**
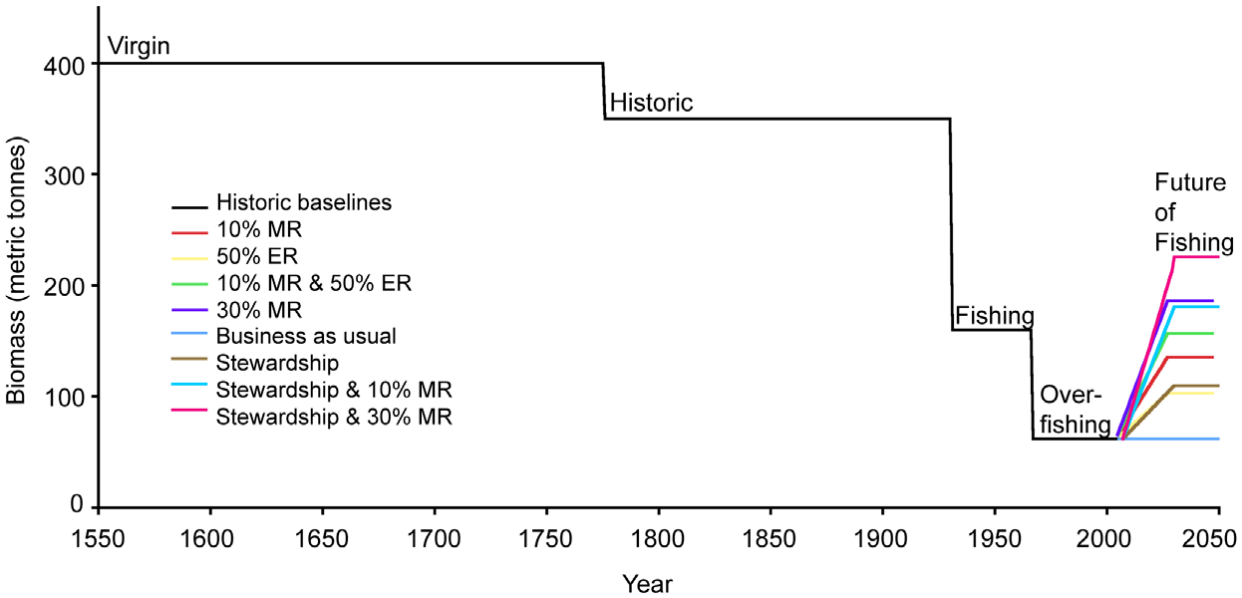
Shifting baselines in lobster abundance in the Juan Fernández Archipelago. Calculated baselines for 1550–2008 and predictions for ‘future of fishing’ modeling scenarios (2008–2050).

Based on our reconstruction of baselines at Islas Robinson Crusoe and Santa Clara, we have estimated lobster biomasses of 400 tonnes for the “virgin” period and 350 tonnes for the “historic” period (Figure 4). Based on stock assessments and reports of CPUE we have estimated average lobster biomasses of 160 tonnes for the “fishing” period and 62 tonnes for the “over-fishing” period (Figure 4). Our model results for the management scenarios predicted lobster biomasses of 62 tonnes for “business as usual”, 140 tonnes for “10% MR”, 105 tonnes for “50% ER”, 160 tonnes for “10% MR & 50% ER”, 200 tonnes for “30% MR”, 113 tonnes for “stewardship”, 185 tonnes for “stewardship and 10% MR” and 235 tonnes for “stewardship and 30% MR” (Figure 4).

### Model Selection

**Figure 5:**
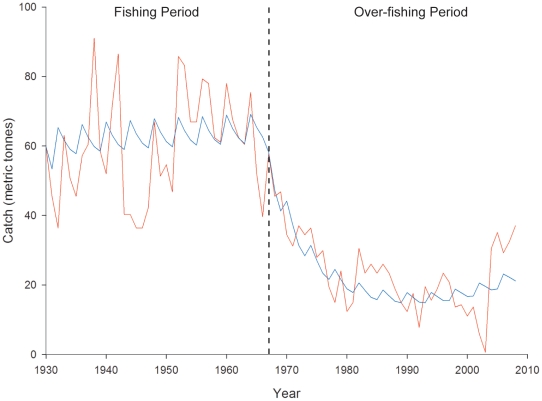
Lobster catch for Islas Robinson Crusoe and Santa Clara. Estimated proportion of Chilean lobster (*Jasus frontalis*) fishery catch from Islas Robinson Crusoe and Santa Clara (red line) with predicted catch from bioeconomic fishery model (blue line) for the “Fishing Period” from 1930–1967 and the “Over-fishing Period” from 1967–2008.

The model that was best able to predict the “fishing” period catch data also best described the “over-fishing” period catch data (Table 3). Inclusion of the annual variability term improved model fit to fishery catch data. For the “fishing” period, the model was able to predict the annual cycles in lobster catch, but not to the same magnitude of fluctuation as was actually observed (Figure 5). For the “over-fishing” period, the model did not accurately predict the frequency of variation in lobster catch, but was able to capture the magnitude of variation for the first part of the catch record and was able to predict the declining trend in lobster catch observed from 1967–1981 (Figure 5). The model does not accurately predict the last seven years (2001–2008) of the “over-fishing” period during which annual fluctuation in catch became highly variable immediately before the fishery was closed in 2004, and then rebounded in the following seasons. The model does however predict an increasing trend in lobster catch at the end of this period, corresponding to the observed catch record (Figure 5). The model is highly sensitive to the catchability term (*q*) as competing models without the term could not be optimized to run for the duration of the period.

**Table 3:** Results of model selection analyses for the “Fishing” and “Over-fishing” Periods.

‘Fishing Period’ (1930–1967)					
Model	Parameters	RSS	K	AIC	Δ*i*
A	ε, q	4283913	2	435.4	47.0
B	ε, P, q	1361955	3	395.0	6.6
C	α, ε, q	1290290	3	393.0	4.6
D	α, P, q	2023225	3	409.6	21.2
**E**	**α, ε, P, q**	**1079734**	**4**	**388.4**	**0**
F	q	3482531	1	425.7	37.3
G	α, q	1433071	2	394.9	6.5
H	P, q	3086683	2	423.3	34.9
‘Over-fishing Period’ (1967–2008)					
A	q	1301423	1	408.2	40.8
B	ε, q	1375611	2	412.4	44.9
C	α, q	913307	2	396.4	29.0
D	α, ε, q	636292	3	384.3	16.9
E	P, q	1301033	2	410.2	42.8
F	ε, P, q	1377988	3	414.4	47.0
G	α, P, q	1146888	3	407.3	39.8
**H**	**α, ε, P, q**	**391 972**	**4**	**367.4**	**0**

RSS represents the residual sum of squares, K represents the number of parameters while AIC refers to the Aikaike Information Criterion value. Model with the lowest AIC value is indicated in bold. Δ*i* is the difference between the AIC value for each model and the model with the lowest AIC value (in bold).

### Model Prediction

**Figure 6:**
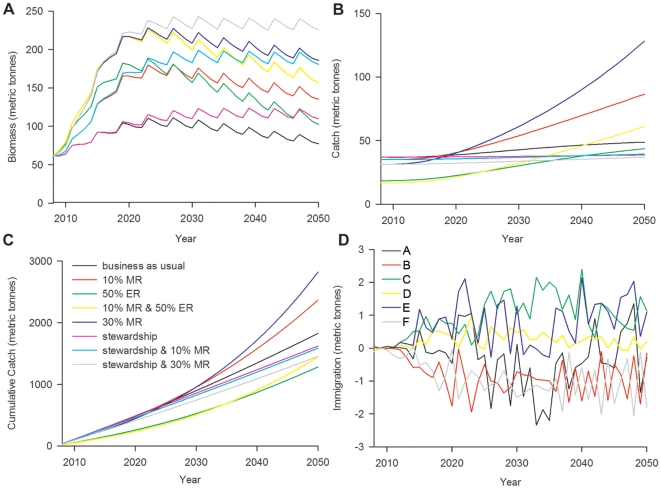
Predicted model results under different management scenarios. **A** - Predicted lobster biomass within the Juan Fernández archipelago for differing management and conservation strategies for the ‘future of fishing’ period from 2008–2050. **B** - Predicted model results for lobster catch in the Juan Fernández archipelago under different management and conservation strategies for the ‘future of fishing’ period from 2008–2050. **C** - Cumulative catch from 2008–2050 predicted from the ‘future of fishing’ model for differing management scenarios. **D** - Predicted lobster spillover by the ‘30% MR’ scenario from the ‘future of fishing’ model. Graph depicts change in population due to spillover in each region (measured as net change in population; positive values correspond to net immigration and negative values to net emigration). Regions A, B and F are closed to fishing with region A located in between regions B & F (see Figure 3 for map). Regions C and E are adjacent to areas closed to fishing, whereas region D does not share a boundary with a closed area (Figure 3).

The “future of fishing” model predicts the “business as usual” scenario to result in the lowest stock biomass at all times, peaking in 2027 at 111 tonnes, then declining slowly to 77 tonnes in 2050 (Figure 6A). The “stewardship and 30% MR” scenario resulted in the highest stock biomass which finishes at 225 tonnes in 2050 (Figure 6A). Scenarios that included the “stewardship” term maintained stock biomass at a relatively constant level after initial growth leveled off (Figure 6A). Scenarios that included the “10% or 30% MR” term showed initial increases in stock biomass, slowly declining after approximately 15 years (Figure 6A). Scenarios that included the “50% ER” term showed a peak in stock biomass after approximately 15 years, finishing with a sharper decline (Figure 6A).

Scenarios that include the “50% ER” term predict the lowest catches throughout the first half of the scenario but finish with greater biomass than other scenarios (Figure 6B) and result in the lowest cumulative catch (Figure 6C). The “10% and 30% MR” scenarios predict the highest catch after approximately 12 years (Figure 6B) and the highest cumulative catch after approximately 20 years (Figure 6C). The “business as usual” scenario predicts relatively constant catch throughout the period, lower than scenarios with the “MR” term and higher than scenarios with the “stewardship” term (Figures 6B). Scenarios that include the “stewardship” term maintain catches at a constant level throughout the period (Figure 6B). All of the scenarios that include the “MR” and “ER” strategies show an exponential increase in cumulative catch at the end the period, while “business as usual” and “stewardship” strategies show a linear growth in cumulative catch (Figure 6C).

### Trade-offs Between Catch and Stock Biomass

The “business as usual” and “stewardship” scenarios resulted in the highest catch initially due to absence of effort displacement, however the lobster population did not increase as quickly as in other scenarios (Figures 6A and B). The reduction in catch observed in scenarios that displace fishing effort through the use of MRs and ER allowed lobster biomass to increase, resulting in a greater rate of population growth. The “10% MR & 50% ER”, “30% MR” and “stewardship & 30% MR” scenarios resulted in the highest growth, but after 2019 the “stewardship & 30% MR” scenario maintained the largest biomass at ∼235 tonnes while the “30% MR” and “10% MR & 50% ER” scenarios declined to ∼200 tonnes and ∼160 tonnes, respectively. The trade-off against the high biomass predicted to occur for the “stewardship & 30% MR” scenario is a reduced growth rate, resulting in less catch (Figures 6A and B). The “30% MR” scenario resulted in the highest catch in 2050 as well as the highest cumulative catch, while also maintaining a high biomass of ∼200 tonnes (Figures 6A, B and C). The “business as usual” scenario resulted in the lowest biomass in 2050 and relatively constant catch throughout the scenario due to low growth associated with low stock biomass (Figures 6A and B).

### Spillover Dynamics

Spillover predicted by the “30% MR” scenario from the “future of fishing” model resulted in net immigration of biomass for fished regions that shared a boundary with the MR (regions C & E) with average annual immigration values of 0.7 tonne and 1 tonne respectively (Figure 6D). Regions protected by the MR that also shared a boundary with fished regions (regions B & F) showed net emigration of biomass with average annual values of −0.8 tonnes and −0.9 tonne respectively (Figure 6D). The fished region that did not share a boundary with the MR (region D) exhibited less variability in spillover with an average immigration of 0.2 tonnes (Figure 6D). The region protected by the MR (region A) that shared boundaries with two regions also protected by the MRs was the most variable with an annual emigration average of −0.3 tonnes (Figure 6D).

## Discussion

### Factors Influencing Model Predictions

A number of different factors, ranging from fundamental aspects of lobster biology to aspects of fishers' behaviour driven by economic necessity, may influence the outcomes of the different model scenarios. The high sensitivity of the model to the catchability (*q*) term suggests that changes to lobster trap technology resulting in greater catchability would have a substantial effect on the dynamics of the system. The small size of the human population, the size of the Juan Fernandez lobster fishery, and the isolation of the archipelago present a unique opportunity to explore these factors and how they might contribute to rebuilding or further decline of the endemic lobster stock.

#### Lobster population connectivity

Based on information about the dispersal of larvae within the Juan Fernández Archipelago and the Islas Desventuradas [62], [67]–[69], in our model we treated Islas Robinson Crusoe and Santa Clara as a closed system. Whereas evidence indicates limited exchange of larvae between Islas Robinson Crusoe - Santa Clara and Isla Alexander Selkirk [67], the dynamics of source and sink populations between the Juan Fernández Archipelago and the Islas Desventuradas are unknown. The west wind drift runs from south to north, which suggests unidirectional flow from Juan Fernández to the Islas Desventuradas. Given this possibility, we suggest that the Robinson Crusoe-Santa Clara lobster fishery should be managed as a closed population.

#### Lobster movement

Knowledge of lobster movement is limited at the Juan Fernández Archipelago, with only reports of changes in depths that traps are set at during the fishing season [49]. In the absence of further information about movements of *J. frontalis*, we use information for *Jasus edwardsii* movement from New Zealand. *Jasus edwardsii* at the Cape Rodney to Okakari Point (Leigh) MR in northern New Zealand exhibited seasonal changes in depth distribution, sex ratio and size frequency which were related to moulting, reproductive and feeding cycles [70]. Additionally, Freeman et al. [21] observed that *Jasus edwardsii* at Te Tapuwae o Rongokako MR in northeast New Zealand were more likely to be re-sighted on the same reef on which they were tagged and unlikely to cross muddy sediments between reefs. The configuration of the MR that protected 100% of one reef resulted in eight times greater abundance than another reef that was 91% protected by a MR [21] indicating that *J. edwardsii* are more likely to “spillover” if MR boundaries occur over continuous rocky-reef habitat. Based on these findings we predict that lobsters in the Juan Fernández Archipelago will respond positively to MR protection when such MRs are sited with due consideration of habitat requirements and natural barriers to dispersal. Further research quantifying larval dispersal patterns, recruitment, lobster movement and location of subtidal reefs and soft bottom at the Juan Fernández Archipelago would be valuable for MR design and model prediction.

#### Climate change, trophic interactions and disturbance

Recent climate change models predict that absolute fishery catch potential will increase slightly (0.5 to 5%) between 2005 and 2055 for the Juan Fernández Archipelago [71]. Trophic structure (and presumably trophic interactions) is not predicted to be affected by climate change as relative abundance of individuals at a given size is not strongly or consistently affected by temperature [72]. New trophic interactions resulting from MR protection could result in higher abundances of lobster predators, however we do not suspect that this will be the case. As reported by fishers, the main predator of lobsters is the octopus [73], but this species is not targeted by fishers. We therefore do not expect the octopus to increase dramatically in abundance with MR protection and in addition, historic states of high lobster abundances in the presence of octopus and other predators have been documented [42]–[45]. Disturbance in the form of increased storm events arising from climate change [72] may impact lobster populations, although given their present depth distributions this seems unlikely. Increased natural disturbance such as storm events may however, contribute to a decrease in fishing activity as the small boats can venture out less often.

#### Illegal fishing

Estimates of illegal fishing activity are, by definition, hard to come by. While illegal fishing will inevitably slow (at low levels of poaching) or even prevent (at high levels) stock rebuilding regardless of the model scenario, measures have been initiated by fishers to prevent them [48]. We suggest that this is because the Juan Fernández Archipelago population and the lobster fishing community itself are both small, members of each are well known, and most families have a mutual interest in fishing. In addition, the geographic isolation of the archipelago offers protection against illegal fishing by “outsiders” which has been shown to break-down co-management institutions in other regions [74]. As such, we doubt that illegal fishing activity will have a significant impact on the model scenario outcomes.

#### Heterogeneity in fishers' responses

The response of fishers, in terms of modification of their own fishing behaviour, will contribute to stock rebuilding or ongoing decline [32]. Individual response among fishers with allocated property (fishing) rights may depend on a number of factors related to livelihood characteristics. It has been shown for fishers in mainland Chile that harvesting decisions may be related to mode of fishing and choices between leaving unfished stock to grow bigger in a subsequent year (e.g., dive fishers for the gastropod “loco”) versus taking stock now to permit immediate investment in new gear (e.g., generalist fishers using nets) [22], [23]. While the responses of the individual lobster fishers may vary according to such factors as personal financial pressure (size of mortgage repayments etc), the fact that all fishers are targeting one species, all are using the same gear, and the fishing community itself is small, leads us to suggest that the fishers' responses will be reasonably homogeneous.

### Historic baselines at the Juan Fernández Archipelago

We have estimated a “virgin” lobster biomass of 400 tonnes. The current stock biomass, estimated at 60 tonnes (15% of virgin), is being maintained through an intensive fishery at the “over-fishing” baseline. There is evidence from New Zealand that historic baselines of lobster abundance can be achieved through the implementation of MRs, on small spatial scales and on timescales observable within a fisherman's lifetime. At Te Tapuwae o Rongokako MR, the subtidal lobster population within the MR has reached density-dependence, such that foraging area has expanded to include a source of algal and invertebrate food sources located on the intertidal platform, an observation not witnessed at neighbouring unprotected locations [75]. This observation is similar to the earliest (pre-exploitation) accounts on the Juan Fernández Archipelago [42], [43] where lobsters were reported in abundance in the intertidal zone, an indication of high densities in the subtidal region. The proportion of suitable habitat that is currently inhabited by lobsters at Islas Robinson Crusoe and Santa Clara is a small fraction of historic accounts and FEK has confirmed greatest depletion of lobsters with proximity to the port, such that the majority of the current catch is now made at the farthest reaches of the archipelago (Table 2). These verbal accounts are supported by reports of CPUE that are three times greater at Isla Alexander Selkirk and more than ten times greater at Islas Desventuradas [49].

### Rebuilding a Fishery

Suboptimal bioeconomic equilibrium is probable in small fisheries with little regulation [76] and may be a legitimate management goal given that it is compatible with the sustainability of the resource [48]. Rebuilding stock biomass has the cost of catching less in the short term. The current “business as usual” management strategy is maintaining lobster biomass at an unproductive level, with catches at a historic low, is highly influenced by annual fluctuations, and has resulted in a reduced CPUE; tenfold less in comparison to the Islas Desventuradas [49]. The enforced closure of the fishery as occurred in 2004 is not a practical management strategy for fishers who already have an annual 4.5 month seasonal closure each year and a high dependence on the resource for their livelihood. However, the higher catches in the two years immediately after this enforced closure suggest that this type of action may be required again in the not too distant future as stock size will not be given a chance to rebuild.

Our modeling results indicate that initial reductions in fishery catch caused by displacement of effort through the use of various management strategies can rebuild stock biomass to levels that can produce catches observed during the “fishing” period. The degree to which the stock biomass increases depends on the amount of effort reduction and/or area closed to the fishery. After approximately 10 and 15 years, catch and cumulative catch are predicted to be equal for both “10% MR” and “30% MR” scenarios respectively, in comparison to the “business as usual” scenario. The “stewardship & 30% MR” scenario rebuilds the stock biomass to the highest level, but provides significantly less catch to the fishery, indicating that this is a more conservation-minded strategy which trades off against economic gain. The “30% MR” scenario shows the greatest potential to increase both catch and cumulative catch which rebuild the stock biomass to approximately 200 tonnes, half of the estimated “virgin” biomass. The degree to which stock biomass is rebuilt will depend on the level of “stewardship” displayed by fishers. Any poaching of the MR will obviously impact its performance to rebuild the lobster population.

It has been suggested that it is for the benefit of the Juan Fernández fishing community that a TAC has not been implemented, as it would disrupt a system of informal traditional tenure [48]. In the absence of TAC, MRs provide an insurance policy for the stock, to ensure that a portion will remain unfished and intact. While a dynamic approach (i.e., rotating the location of the MR) may benefit some trophic groups [77], we suggest that in the present case where the focus is on a “sessile” species, or at least a species with low mobility, a static MR approach will be more beneficial in line with findings elsewhere [11]–[14]. This approach also has the benefit of being easier to delineate (on maps and with coastline markers or buoys) and easier to enforce. The proposed MR location was sited by fishermen as it will displace minimal fishing effort (most depleted of lobster) and so that it can be observed by village members.

Despite the obvious long-term (sustainability) benefits of a co-management and stewardship strategy, implementing fisheries management practices where this phenomenon is observed is often the real challenge [10], [78]. Increasing ownership and implementing community-based co-management of the fishery catch has been shown to increase compliance, promote conservation values and transfer the burden of management and enforcement by using a bottom-up approach [22], [24], [25]. Our conversations with fishers indicated a sense of disparity in the historically low catches in comparison to higher catches from the “good old days”, which has been shown to foster a greater willingness to change existing practices [22]. Recent reports [48] indicate that fishermen have insisted on the need for a regular presence of the fisheries authority on the islands to improve the quality of landing statistics and the enforcement of size regulations. As a result, a voluntary logbook program has been started to record spatial CPUE data, which is a good indicator of stock abundance [48]. Following our conversations with fishers where we introduced the idea of MRs to many of them for the first time [79], it has been reported that the lobster fishers' syndicate is trying to create a MR extending to 10 nautical miles around the islands with the ultimate goal of excluding mainland-based fishing fleets [48]. Based on recent participation and demand for inclusion in management decisions by lobster fishers to employ regulatory, monitoring and conservation initiatives we believe that our proposed co-management strategy to determine the level to which stock biomass is rebuilt is realistic and compliance would be high. The isolation of the Archipelago makes it unlikely that “roving bandits” from other fishing communities [74] pose a threat.

### The Use of FEK and Historic Sources

Our approach, employing the bioeconomic fishery model for the long time period we examined has strength in its ability to place current biomass stock in the context of virgin biomass. Given that estimates of lobster biomass throughout time are patchy, often qualitative and not spatially resolved, FEK and historic sources permit investigation of the current state of resource exploitation. We do not seek to estimate how much lobster can be taken today without causing the population to collapse, that is the realm of stock assessments. Our aim is to show how trading off some catch today will not only provide greater landings and higher CPUE in the future, but also provide a whole suite of conservation and ecosystem-based management achievements through MR implementation, while giving control and responsibility of the resource to the fishers. The voluntary CPUE logbook program that is now in place [48] will provide an accurate, spatially resolved indicator of abundance to allow for better informed management and conservation decisions in the future.
